# Microcomputed X-Ray Tomographic Imaging and Image Processing for Microstructural Characterization of Explosives

**DOI:** 10.3390/ma13204517

**Published:** 2020-10-12

**Authors:** John D. Yeager, Lindsey A. Kuettner, Amanda L. Duque, Larry G. Hill, Brian M. Patterson

**Affiliations:** 1 High Explosives Science and Technology, Los Alamos National Laboratory, Los Alamos, NM 87545, USA; aduque@lanl.gov (A.L.D.); lgh@lanl.gov (L.G.H.); 2 Engineered Materials, Los Alamos National Laboratory, Los Alamos, NM 87545, USA; lkuettner@lanl.gov

**Keywords:** microstructure, explosives, microcomputed tomography, dual energy, segmentation, composites, polymer matrix composites

## Abstract

Microstructural characterization of composite high explosives (HEs) has become increasingly important over the last several decades in association with the development of high fidelity mesoscale modeling and an improved understanding of ignition and detonation processes. HE microstructure influences not only typical material properties (e.g., thermal, mechanical) but also reactive behavior (e.g., shock sensitivity, detonation wave shape). A detailed nondestructive 3D examination of the microstructure has generally been limited to custom-engineered samples or surrogates due to poor contrast between the composite constituents. Highly loaded (>90 wt%) HE composites such as plastic-bonded explosives (PBX) are especially difficult. Here, we present efforts to improve measurement quality by using single and dual-energy microcomputed X-ray tomography and state-of-the-art image processing techniques to study a broad set of HE materials. Some materials, such as PBX 9502, exhibit suitable contrast and resolution for an automatic segmentation of the HE from the polymer binder and the voids. Other composite HEs had varying levels of success in segmentation. Post-processing techniques that used commercially available algorithms to improve the segmentation quality of PBX 9501 as well as zero-density defects such as cracks and voids could be easily segmented for all samples. Aspects of the materials that lend themselves well to this type of measurement are discussed.

## 1. Introduction

A characterization of the microstructure of high explosives (HEs) is important for constructing accurate mesoscale simulations; understanding processing–structure–property relationships, which influence initiation and detonation behavior; and quantifying production parameters (i.e., lot-to-lot variations). The microstructure of HE, and in particular grain size and size distribution, has been shown to affect shock sensitivity [1], detonation velocity [2], critical diameter [3], detonation front curvature [4], and corner turning [5]. Other relevant features of the microstructure include, but are not limited to, grain morphology [6], distribution of constituents [7], void size distribution [8], shape of void [9], and damage [10]. Further, multiple categories of explosives exist and have their own unique behavioral traits. Plastic-bonded explosives (PBX) are highly-loaded polymer matrix composites that can be formulated in a wet slurry process and pressed or formulated as cast-curable materials [11,12,13]. The polymers (“binders”) can be inert and only serve to enhance stability and machinability, or they can be somewhat energetic themselves and contribute to the detonation energy [14]. Melt-castable explosives can be a single low melt temperature material (e.g., trinitrotoluene) or a mixture of explosives, where at least one component is melt-castable so it can serve as a binder (e.g., Composition B) while imparting high detonation energy [15]. Some explosives are simply used as a powder or pressed into a pellet without a binder (“neat”) [16]. As is the case in many materials, quantifying all microstructural features with a single technique is difficult if not impossible; since most explosives are relatively fragile, not to mention hazardous, obtaining accurate measurements is especially challenging.

Historically, most of the microstructural characterization of HE has been done in 2D with optical microscopy or similar methods [17,18,19]. The data can then be used as a starting point for simulations either directly (e.g., digitizing 2D images) or indirectly (e.g., numerically generating microstructures that match the measured particle size and morphology) [6,20,21]. These approaches have advantages in terms of experimental and computational efficiency, but they also have limitations in their ability to quantify and accurately assess damage and other microstructural features across representative amounts of material. Probably the most commonly used 3D characterization technology at this scale is X-ray microcomputed tomography (µCT), and several recent works have explored the feasibility of using µCT to comprehensively characterize explosives or explosive-like surrogates [22,23,24,25]. While µCT can be an expensive technique (i.e., in cost, data, and time), it is often worth the effort because it can quantitatively measure voids, particles, cracks, and other features, and it can also be used to track the changes of these features due to external insult [26,27,28]. For traditional materials, µCT is often used as input data to create 3D meshes of materials as a realistic starting point for mesoscale modeling. For an accurate mesh construction of heterogeneous materials, the various constituent phases must have sufficient X-ray contrast to enable segmentation. 

Unfortunately, most explosive constituents have little X-ray contrast, mainly due to similar composition and density. These difficulties are exacerbated in cases where the material is close to theoretical maximum density, or when the composite explosive is very highly loaded (>90% HE). In short, measurement of “real” explosives with µCT has been generally limited to detecting voids or cracks [25,29,30], and fully segmentable microstructures have only been obtained by using engineered or optimized samples [23,24,31]. An analysis of voids and cracks is still useful for safety and initiation modeling [8,32,33], but full spatial information would greatly enhance these efforts while also enabling real microstructures (i.e., accurately described crystal and binder distribution) to be used for mesoscale simulations (e.g., [34]).

In order to obtain optimized images for typical (non-HE) samples, state-of-the-art instrumentation relies on a variety of techniques including automated ring artifact removal, multiple background collections to remove instrument drift, and automated filtering to reduce beam hardening artifacts. To optimize the sample contrast, instruments are also capable of fine-tuning the X-ray energy through source energy selection, filter application, and dual-energy imaging to improve the contrast in similar atomic number samples. After the data are collected, modern software packages also invoke a plethora of imaging filters (i.e., median, unsharp, edge-preserving to name a few) that are used to smooth out noise and enhance edges. Finally, segmentation techniques have been developed that are as simple as choosing a grayscale range, to watershed, auto intensity correlation, and state-of-the-art texture-based machine learning. Determining the correct combination of all of these parameters is a difficult and time-consuming challenge, and this results in a final image quality that is much an art as it is a science [35].

Here, to evaluate the state-of-the-art in laboratory-based µCT for HEs, we imaged ten different HE materials at their typical density and composition. The chosen materials are of interest to a wide variety of modern explosive and weapons applications. This study resulted in a library of high-quality images for future modeling and morphological studies. Additionally, a variety of techniques were investigated, with varying success, in postacquisition image segmentation. The human brain is very well developed in defining the edge boundaries between crystals and the binder. However, HE and HE composite materials represent about as tough a materials science challenge in segmenting images as any material system. HE crystals have densities and atomic composition that are nearly identical to the modern binders used to hold them together. Initially, we discuss the most conducive PBX system for µCT imaging, then evaluate six PBX materials with various polymer binders, and finally we measure HEs with no polymer binder. A variety of image acquisition and post-processing techniques will be demonstrated, including optimized X-ray energy, dual energy imaging, filtering, and advanced segmentation approaches. While dual energy X-ray imaging is used in security applications to detect the presence or absence of explosives, to our knowledge, this is the first presentation of the use of dual-energy µCT to improve the contrast in the study of the microstructure within HE composite systems.

## 2. Materials and Methods

### 2.1. Materials

A full list of the ten HE materials studied here is given in Table 1. The materials variously contained the explosives cyclotetramethylene-tetranitramine (HMX), cyclotrimethylenetrinitramine (RDX), triamino-trinitrobenzene (TATB), and trinitrotoluene (TNT). Six of the materials were traditionally-manufactured PBX formulations, with the HE filler being around 95% by weight and the remainder a polymer binder. Polymers included the block copolymer Estane, the fluropolymer Kel-F, the fluoropolymer Viton A, hydroxyl-terminated polybutadiene (HTPB), and nitrocellulose. The seventh PBX formulation, HMX-HTPB, was similar to a previously studied formulation that had been shown to be highly measurable with µCT. Two formulations were melt-castable, where TNT was used as the matrix that contains other HE crystals. The tenth material was TATB without a binder. These materials were chosen to span a variety of HE formulation conditions, processing methods, particle size distributions, and binder materials.

Measurement specimens were primarily used as-supplied from the appropriate production agencies and were made by compacting raw materials into 3.2 mm diameter × 6.4 mm tall right circular cylinders. Compaction into solid samples was achieved by die-pressing at ambient temperatures to an approximate pressure of 41.5 kPa in a hardened steel die. Composition B and Octol are traditionally melt-cast to shape, but for better comparison at this small sample size, premixed materials (flakes) were pressed identically to the PBX samples. Final part densities for all pressed samples were within the specification for their typical application. The HMX-HTPB sample required a curing process for the binder to become stable, and so it was cast as a large block instead of pressed; 3 mm diameter × 3 mm tall cylindrical samples were bored out of the block for measurement.

It should be noted that these sample sizes were optimized for the µCT measurements, and while they are representative of processes used to make full-scale HE charges, this probably introduced higher damage to the material than would be expected at larger scales. The unintended benefit of this was that microstructural defects such as cracks and voids would be more ubiquitous than in traditionally manufactured material, highlighting the utility of the µCT technique for characterizing such defects. Some care should be taken before comparing the results found here for any given material to “bulk” or typical large scale material microstructure.

### 2.2. Computed Tomography

Three Micro X-ray CT images (µCT) were collected of many of the samples using an Xradia Versa 520 MicroXCT (Carl Zeiss X-ray Microscopy Inc., Pleasanton, CA, USA). A high resolution image (≈0.8 micrometers voxel size) was collected of each sample, and two lower resolution images were collected on many of the samples to explore the possibility of using dual-energy imaging to improve imaging contrast. The system uses a microfocus X-ray source with a transmission tungsten anode. A scintillator converts the X-ray photons to visible light photons and is mounted on the front of the objective, which then transmits the optical photons to the 1k x 1k piezoelectrically cooled detector. The 20× objective was used, except as noted, with no source filter; the sample was rotated 360° while imaging, and a binning of 2 was used on the detector. The high-resolution image of each sample was collected, and their imaging conditions are outlined in Table 2. Each used a peak potential of 50 kV and a total of 4 W power. The images were reconstructed using the TXMReconstructor (Carl Zeiss X-ray Microscopy Inc., Pleasanton, CA, USA) and rendered and quantified using Avizo Inspect 2020.1 (Thermo Fisher Scientific, Hillsboro, OR, USA). The images were smoothed with an edge-preserving filter and segmented based upon the grayscale values; the crystals, binder, and voids were separated and quantified.

## 3. Results

### 3.1. Plastic-Bonded Explosives

**Figure 1:**
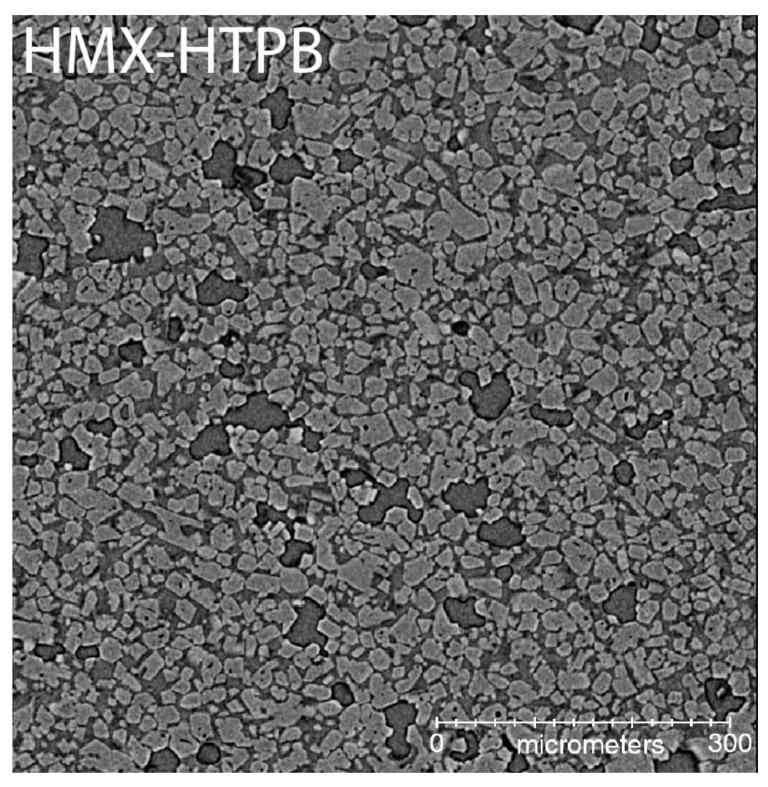
HMX-HTPB formulation imaged with high resolution X-ray microcomputed tomography (µCT). The crystals are the light gray, the binder is a medium gray, and the voids are the darkest gray. Some phase contrast (light–dark halo) is seen around the voids. This is minimized by reducing the distance from the sample to the detector scintillator.

Figure 1 shows a typical µCT for the HMX-HTPB sample. The lighter material is the HMX, while the black constituent is void, and the intermediate grayscale material is the binder. This contrast scheme is consistent for all the CT images shown here, as the grayscale value is lighter as density increases. This particular HMX-HTPB formulation was manufactured with Class V HMX only, meaning that effectively all HMX particles were smaller than 45 µm in diameter as measured with sieve cuts. The resulting microstructure is an approximately monomodal distribution of HMX, and the binder appears to be reasonably well intermixed but definitely has some regions of higher concentration. As mentioned earlier, single energy µCT measurements of this class of material have been proven sufficient for material segmentation to create meshes for modeling, and it represents a “gold standard” for PBX microstructure characterization [23].

**Figure 2:**
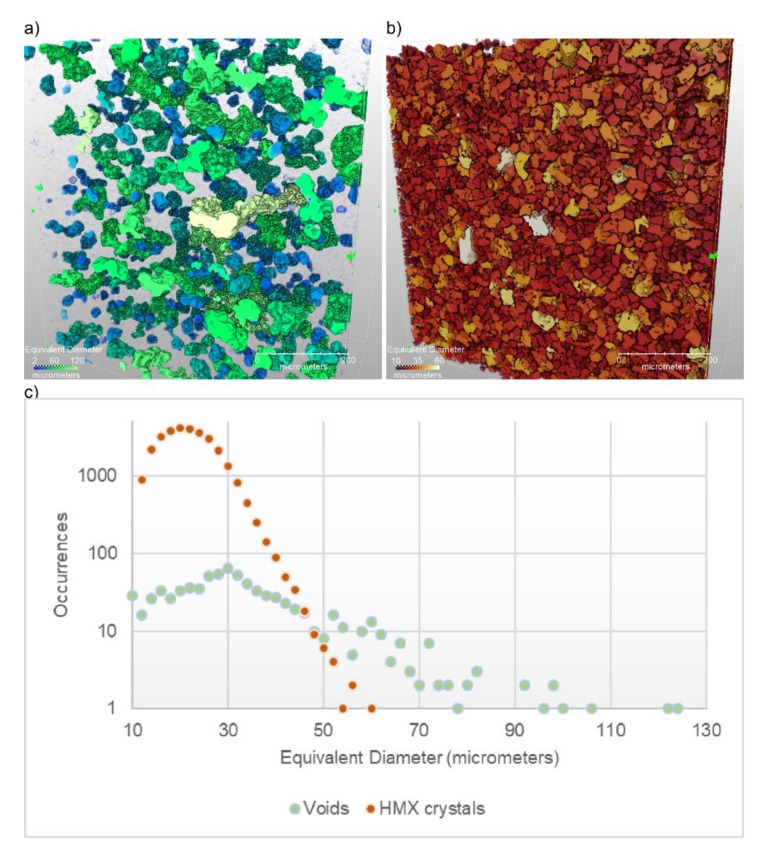
3D rendering of the voids (**a**) and the crystals (**b**) within the HMX-HTPB plastic-bonded explosives (PBX) system. The voids and the crystals are colored by their equivalent diameter with the graph (**c**), a plotting of a histogram of their sizes. The equivalent diameter is the diameter of a sphere with the same volume as the object.

Figure 2 shows the results of the segmentation of the HMX-HTPB system. The densities of the HMX (1.9 g/cm^3^) and the HTPB (0.9 g/cm^3^) are sufficiently different that this system can be segmented for voids, crystals, and binder. Upon data collection and importing into Avizo, an “edge-preserving smoothing” filter was used with values of (stop = 1000, step = 100, contrast = 100,000, and sigma = 0). For this filter, a range of decades (10^3^ to 10^6^) of “contrast” were explored and optimized, while the other values were held constant. Then, a combination of “magic wand” and simple grayscale threshold was used to create the segmented image.

**Figure 3:**
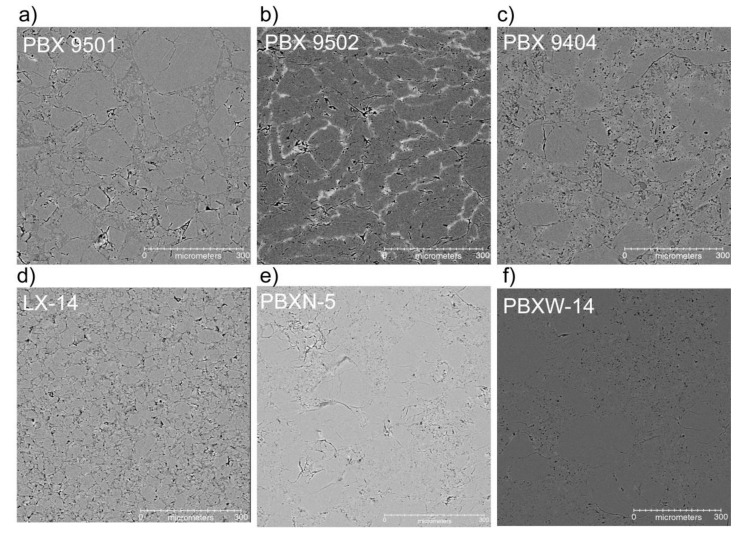
Reconstructed µCT slice images from the six plastic-bonded explosive materials. PBX 9501 (**a**), PBX 9502 (**b**), PBX 9404 (**c**), LX-14 (**d**), PBXN-5 (**e**), and PBXW-14 (**f**). The images are through the center of the high explosive cylinder and are nearly completely free of all imaging artifacts.

Figure 3 compares the other six PBX formulations with a single reconstructed slice taken in one glance, with detailed comparisons being shown in later figures. All images have only been altered with minor brightness and contrast adjustments. While each material has approximately the same concentration of the binder, there are obvious differences in image contrast between the various materials. This is likely due in large part to crystal size distribution and similarity in density between the crystals and binders. HMX has a density of 1.905 g/cm^3^ and TATB has a density of 1.937 g/cm^3^. Viton A has a density of ≈1.8 g/cm^3^, Estane is ≈1.1 g/cm^3^, and the plasticized binders in PBX 9501 and PBX 9404 are ≈1.2 g/cm^3^ and 1.5 g/cm^3^, respectively. The relatively large density differences between HMX and the non-Viton binders probably contribute to the clarity of the images. For PBX 9502, the Kel-F binder is actually slightly higher in density (2.02 g/cm^3^) than the TATB crystals, meaning it shows up as a lighter grayscale value. Both PBX 9501 and PBX 9502 were studied in more detail here to attempt to segment the binder, crystals, and voids.

#### 3.1.1. Segmentation of PBX 9501 and PBX 9502

PBX 9501 and PBX 9502 are the most commonly studied PBX materials at Los Alamos, and as mentioned earlier, there exist many research efforts to link experimental measurements to theoretical and computational studies at multiple time and length scales. Therefore, an effective method for the segmentation of the PBX materials into their constituent parts would be of high value. To our knowledge, phase segmentation based on direct microstructural interrogation has never before been accomplished in these materials.

**Figure 4:**
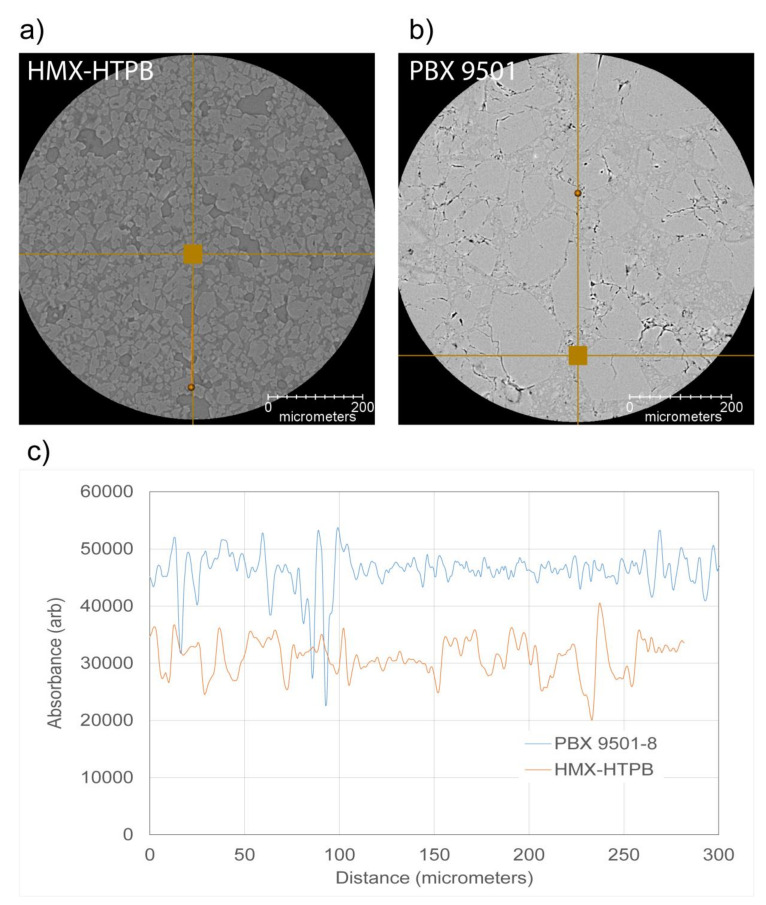
Reconstructed slice through HMX-HTPB (**a**) and PBX 9501 (**b**) composite comparing the gray scale differences between the crystals and the binder. Line profiles extracted from the images show the improved signal-to-noise in the HMX-HTPB sample (**c**).

Segmentation of the reconstructed PBX 9501 was attempted with a variety of techniques. As mentioned before, our human brains are quite adept at picking out patterns within the images and detecting the crystals; however, a line profile of the grayscale values through the dataset only shows noise when passing through crystals and binders. Upon importing into Avizo, the data were filtered with the edge-preserving smoothing filter (90k as the optimal contrast value). At this point, the segmentation for voids is a relatively straightforward grayscale selection. However, the minor density difference between the crystals and the binder means that they cannot be adequately segmented based on a single image. Figure 4 shows a single reconstructed slice of both HMX-HTPB and PBX 9501 with a line profile through the composite. As can be seen, the grayscale values between the crystals and binder are adequate for the segmentation of the HMX-HTPB seen in Figure 2, but they are not adequate for the PBX 9501; more advanced data acquisition and processing are required.

**Figure 5:**
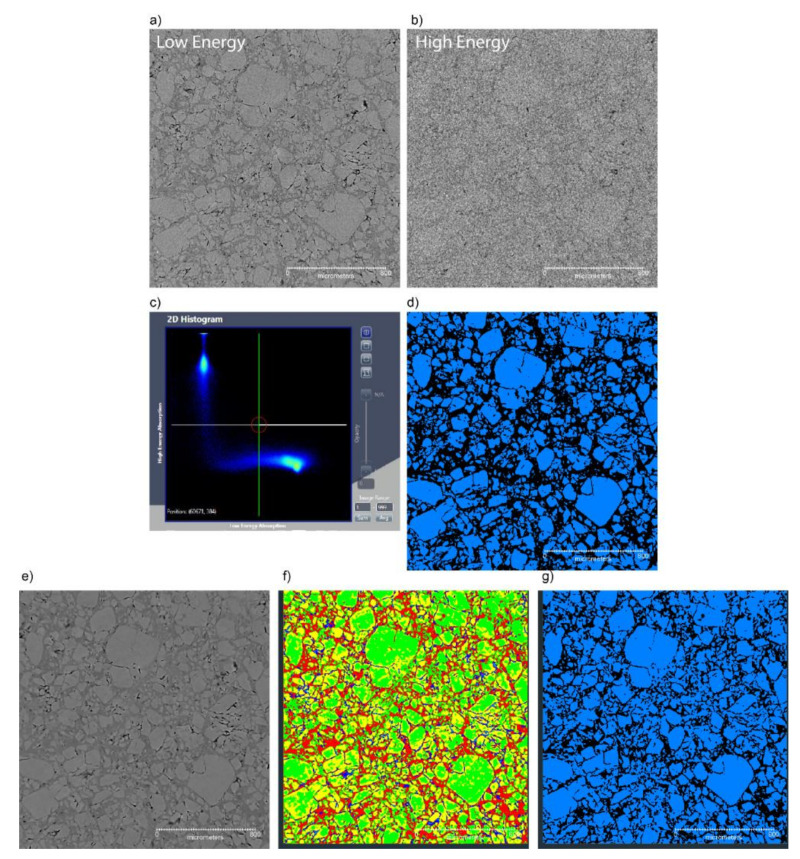
Segmentation attempts of PBX 9501 images using two different energies for the dual energy imaging technique, where (**a**) and (**b**) are the low energy and high energy reconstructed slices, and (**c**) is the 2D correlation plot of the high and low energy grayscale values used to select the crystals (**d**). The blob in the lower right is two unresolved spots; one represents the crystals, and the other is the binder; (**e**) is the output subpixel aligned low energy (SALE) image, sharpened, then segmented using the auto intensity calibration (**f**), and then finally segmented for the crystals (**g**).

In order to try to improve the grayscale values between the crystals and the binder, most of the samples were imaged using a dual-energy modality. This modality images the samples at two different X-ray energies. The sample must fit within the field of view of the image; therefore, these images were at a lower resolution than the images shown in the previous section at 3.78 µm. The two images rely on the difference in absorption processes between the photoelectric effect at low energies and Compton scattering at higher energies. To obtain the greatest separation in X-ray absorption, the two images of the samples were run at low energy (30 kVp) and again with the source at high energy (160 kVp) and a filter (LE5) to have an average photon energy of ≈70 kV, as seen in Figure 5a,b. These two images were then imported into the Zeiss Dual-Energy Contrast visualizer software. The noise reduction filter was applied once to the low energy image and twice to the high energy image. The filtered data were plotted as a 2D histogram as seen in Figure 5c, which shows a plotting of the grayscale value of the low energy image versus the grayscale value of the high energy image for each voxel. Four “hot spots” are noted on the plot. They represent the air around the sample (not shown, as the reconstructed slices are zoomed in to aid the reader in seeing the crystals), the interface of the air to the sample and the voids (both on the upper left of the plot), and the voxels representing the grayscale value of the crystals and binder, which are represented by the hot spots in the lower right quadrant of 5c.. Ideally, the points on the histogram are completely separated from each other; they were selected using the tool and then exported as a.txm file for plotting in a rendering package. For this study, there is some overlap in the regions representing the crystals and the binder that were selected and exported. A slice through the image of the crystals is shown in Figure 5d. This technique measures the crystals as occupying a percent volume of 57.6%.

A second composite dual-energy image was exported out of the dual-contrast visualizer as a subpixel aligned low energy (SALE) image. This image was then imported into Avizo, and a sharpening filter was applied. This 3D image was then cropped to only contain the sample and segmented for five regions using auto intensity correlation (Figure 5f). Two of these regions represent the crystals and the interface to the crystals to the binder (Figure 5g). This technique measures the crystals as occupying a percent volume of 57.2%. This excellent agreement between the two techniques seems to indicate their robustness. The sample contained 3% of the measured void volume.

A theoretical maximum density (TMD) of the sample of PBX 9501 should be 92.7% by volume HMX, 3.9% by volume Estane, and 3.3% by volume plasticizer. Using either segmentation technique, the calculated crystal content underestimates the reality by about a third. The most likely reason for this underestimation is that a significant fraction of the HMX crystals are too fine to be adequately distinguished from the binder given the spatial resolution. The HMX size distribution is approximately 3:1 coarse to fine, where “fine” includes any particles under 45 µm equivalent diameter [36]. The voxel size of the dual-energy approach was 3.78 µm, with a spatial resolution of ≈12 µm (assuming a 3 voxel requirement to define an edge). This means that the largest of the fine crystals detected would only be 10–12 pixels across. Moreover, many of the fine crystals are in fact well below 45 µm [37]. It is difficult to distinguish crystals at only a few pixels across with any confidence. Another contributing factor could be that the crystal boundaries are not sharply defined from the binder due to gradient composition or intermixing, which is known to be possible in PBX 9501, albeit at finer scales [38]. Gradual changes in grayscale values make it more difficult for the software to distinguish edges, possibly leading to the underestimation of true crystal volume.

Unlike PBX 9501, segmentation in PBX 9502 was relatively straightforward because the binder shows up so clearly in the images (Figure 3). This is probably due to two effects. First, the Kel-F binder has chlorine and fluorine while the TATB is only organic (CHNO). Second, the Kel-F is believed to preferentially form shells around binder-poor TATB agglomerates during the slurry formulation process rather than evenly coating the TATB crystals. While those shell-agglomerate particles (also known as molding prills) are typically at the millimeter scale during shipping and handling, compacted samples of this type of material are known to retain some residual prill structure even when pressed to high density [39,40,41]. The dark clumps in between the binder are not likely to be individual crystals of TATB, since the TATB used in PBX 9502 is effectively identical in particle size to the neat TATB studied here (≈20 µm), which is presented later. The dark regions here are likely agglomerates of TATB crystals, indicating that there are multiple length scales of interest when characterizing the PBX 9502 microstructure; that is, the individual crystal scale and the crystal agglomerate/molding prill scale. This finding also reinforces some recent modeling approaches in the literature that account for multiple length scales to match experimental data [42,43].

**Figure 6:**
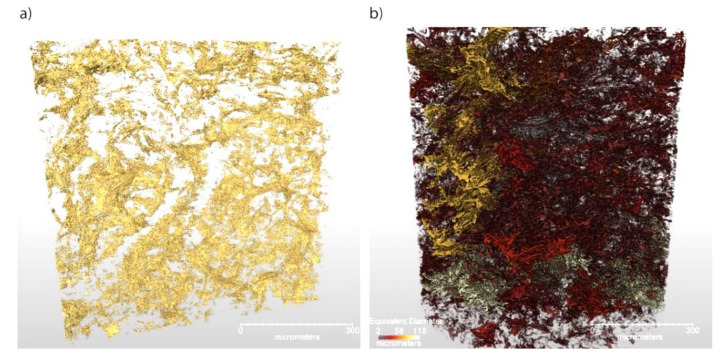
Segmented partial volumes of the measured PBX 9502 microstructure, showing binder distribution (**a**) and void size distribution (**b**). Some concentrations of voids can be seen, probably indicating cracks or TATB crystal boundaries.

The contrast in the PBX 9502 sample is such that the segmentation into crystal, binder, and voids was possible using the machine software. To our knowledge, this is the first time that a microstructural segmentation of such a highly loaded explosive has ever been accomplished. Figure 6 shows renderings of the binder distribution and the void distribution, with the latter color-coded by equivalent diameter. Among other measurements, this type of segmentation enables calculation of the relative volume fractions of each constituent. Of course, the calculation is limited by the voxel size of the measurement, but rough estimates are easily obtained. Here, the segmentation calculates the binder percent as 4.68% by volume, which can be converted to a weight percent of ≈4.9%, essentially identical to the desired formulation. The void content is measured to be 3% with a standard deviation of 1%. Based on the pressing conditions, the estimated void content for this formulation is ≈2.5% and is thus within the standard deviation for PBX 9502.

#### 3.1.2. Comparison of Three Highly-Loaded HMX Formulations

**Figure 7:**
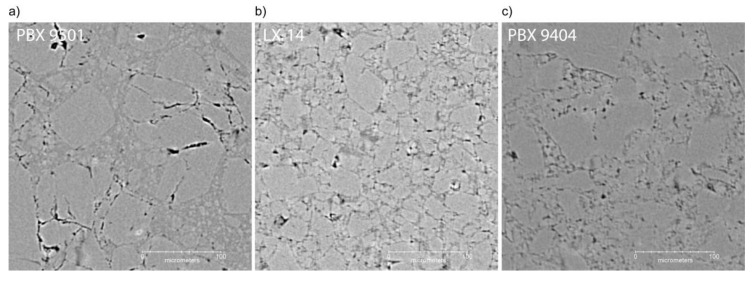
µCT reconstructed slices of LX-14 (**a**), PBX 9501 (**b**), and PBX 9404 (**c**) microstructures.

Figure 7 shows selected regions of three similar PBXs to highlight the influence of microstructural features on the usefulness of this technique. Specifically, these three materials have slightly different crystal-binder density ratios, HMX content, and HMX particle sizes. LX-14 is shown in Figure 7a, having a very broad particle size distribution, and perhaps because of this it seems to have a slightly higher concentration of voids than the other PBXs. PBX 9501 (Figure 7b) is similar to LX-14 in that it has nearly identical HMX content and uses an Estane binder, but the binder is plasticized and the particle size distribution is a more well-defined 3:1 coarse-to-fine ratio. PBX 9404 (Figure 7c) is similar to PBX 9501 in HMX particle size distribution but has a nitrocellulose binder that is closer in density to the HMX crystals than is to the Estane. In both of the latter PBX materials, the large HMX crystals often present internal cracks, likely due to their brittle nature, which manifests when absorbing a high amount of the pressure during the compaction process.

The differences in the ability of dual-energy μCT to characterize these materials is instructive. The instrument resolution for these three samples was identical. The microstructure “resolution”, which is a function of the material properties and the instrument resolution, appears to be better for LX-14 and PBX 9501 than PBX 9404. The most likely contributor to this is the binder-HMX density differences, where PBX 9404 has the smallest difference. We also note that HMX is shown to have the ability to intermix in nitrocellulose [44], so the material processing conditions may also lead to rounded crystal edges or HMX-nitrocellulose intermixing, further obfuscating the image. The PBX 9501 appears to have the most well-defined microstructure, possibly due to the larger particle size distribution as compared to LX-14.

### 3.2. HE Materials without a Binder

**Figure 8:**
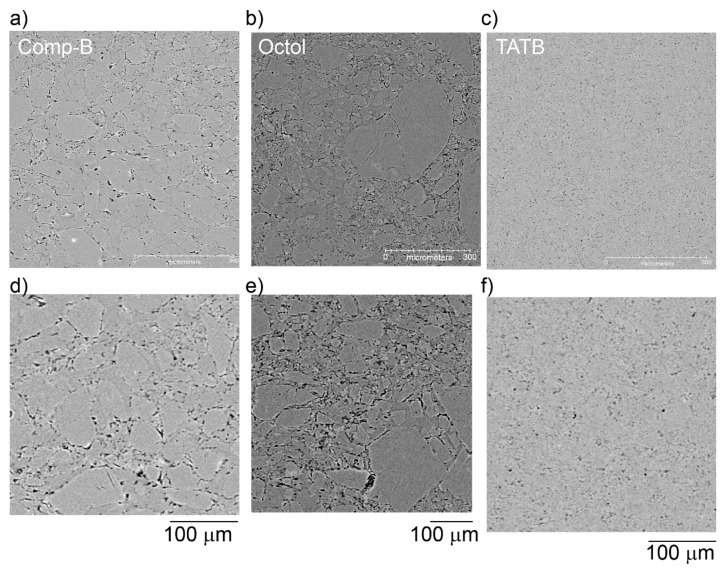
Typical µCT reconstructed slices of the microstructure for Composition B (**a**,**d**), Octol (**b**,**e**), and TATB (**c**,**f**) at two fields of view.

Figure 8 compares the three HE materials that do not have a polymer binder. Composition B (Figure 8a,d) and Octol (Figure 8b,e) use TNT as the matrix, with RDX and HMX filler HE respectively, and some differentiation between the TNT and the filler HE can be seen. The TATB (Figure 8c,f) images are not particularly easy to interpret by eye, but some grain boundaries can be observed, and certainly the distribution of voids can be characterized. The average particle size calculable for TATB in this case is on the order of 20 μm, which is in a similar size regime to TATB specifications and other studies [39,45,46]. This small particle size reinforces the PBX 9502 findings, where crystal agglomeration between distinct binder regions must be happening because their sizes are much larger than the small crystals found here.

**Figure 9:**
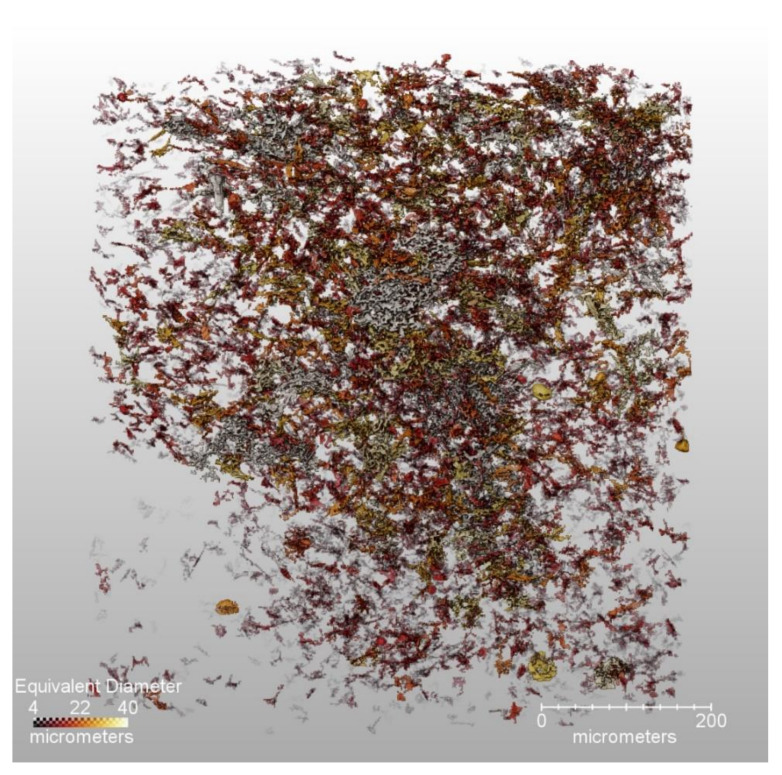
3D rendering of the voids within the Composition B explosive, colored by equivalent diameter. The largest voids are about 40 µm in equivalent diameter, but in reality, they are mostly flat planes throughout the sample. The voids are not evenly distributed and occupy 2% of the total volume.

For the two TNT-based samples, the larger crystals can be distinguished from the matrix by eye, but the only segmentable feature is the void structure. The void structure of the TATB sample is also easily measured. Figure 9 shows the void structure of Composition B. Within the limits of spatial resolution, some of the “voids” appear as elongated planes and are probably more accurately described as cracks. We note that the processing method used here (pressed to shape rather than melt-cast to shape) may have some influence on the measured properties.

## 4. Discussion

### 4.1. New State-of-the-Art for Full PBX Segmentation

This comprehensive effort to measure microstructural features in HEs with state-of-the-art laboratory µCT equipment and software demonstrates some important advances but also shows just how far is left to go. From a mesoscale modeling perspective, fully phase-segmentable datasets are needed for direct implementation into simulations. For the PBX materials, this means a complete segmentation of each constituent material, along with the voids. For the HEs without polymers, ideally both voids and grain boundaries would be segmentable. Here, we have successfully segmented two of the ten measured HEs to this level of precision. While success with the HMX-HTPB formulation was expected as it was characterized before, the full segmentation of PBX 9502 is very exciting. To our knowledge, this is the first time ever that a “real” (i.e., highly loaded, nonengineered, nonoptimized) explosive was segmented into constituent phases. A third HE, PBX 9501, was partly segmentable by using two different analysis techniques, especially the larger crystals, but work is underway to develop other segmentation techniques to be able to fully use current µCT scans. It is possible that a focused numerical effort, using machine learning or other advanced segmentation technology, could fully segment the PBX 9501 dataset. The segmented µCT data for these three HE materials are available for download in the Supplementary Materials to aid in future mesoscale simulations.

### 4.2. Measurements of Voids and Implications for Modeling

For the other materials, segmentation was generally limited into voids vs. solids. Void size distributions do occasionally reveal some interesting features of the microstructure, such as grain boundaries or cracks, and there are many mesoscale modeling approaches that could use this data as is. However, the obvious nonhomogeneity of the void distributions (Figure 2, Figure 6 and Figure 9) also has important implications for HE detonation modeling at the continuum scale.

For modeling to be tractable for computing detonation in charges that are very large compared to the HE microstructure, detonation reactive flow models (RFMs) must treat the explosive as a continuum. Accordingly, they utilize a reaction rate law that is expressed in terms of continuum variables. Nevertheless, in order to capture underlying heterogeneous reaction mechanisms, continuum reaction rate laws must suitably reflect the microscopic driving mechanisms. The exercise of distilling the essence of heterogeneous reaction mechanisms into a continuum rate law is called *homogenization*.

The only formal homogenization problem that has been performed to date considers a collection of randomly-placed hot-spot nucleation sites within a considered fluid element. Just as for other multiphase fluid models, the element is assumed to contain enough nucleation sites to be statistically representative, yet still small compared to the reaction zone thickness. Spherical burn waves grow from the nucleation sites, consuming the interstitial reactants: This early burn-phase comprises so-called “hole burning” or “progressive burn,” terminology borrowed from the propellant community. The essential problem is how these burn waves coalescence. The late consumption of material has a “grain burning” or “regressive burn” morphology, and intermediate phases are transitional forms [47,48,49].

It is interesting and perhaps a bit surprising that the problem has a simple analytic solution, which was first discovered and derived in essentially maximum generality by Kolmogorov in 1937 —the original application being heterogeneous phase changes in metals. Within a few years, a subset of the same result (i.e., the impulsive nucleation limit) was independently derived by Avrami, using an alternative nonstatistical approach [50]. In the field of material science, the result is now known as the Johnson–Mehl–Avrami–Kolmogorov (JMAK) equation.

The JMAK model was first used in the context of detonation by Hayes, who independently derived the impulsive nucleation limit in 1983 [51]. The JMAK model was first invoked as an RFM component by Nichols, who once again independently derived the result in 2002 [52]. Nichols called the result the statistical hot spot (SHS) model, which is the name by which it continues to be known in the detonation community.

The SHS prescription is now used by several modern RFMs, including the Los Alamos Scaled Uniform Reactive Flow (SURF) model. When Menikoff and Shaw set about to calibrate and validate SURF [53], they observed that the 2D SHS variant (i.e., expanding and coalescing circles or parallel cylinders) yielded better agreement with detonation test data than did the 3D variant (i.e., expanding and coalescing spheres).

The reason for this curious observation has been a matter of some speculation; however, the void distribution shown in Figure 6b illustrates why such behavior is perhaps to be expected. Here, it appears that voids tend to concentrate at those crystal interfaces into which the binder has been unable to flow. The result is a collection of sheet structures resembling a bowl of cornflakes. As these are essentially 1D structures embedded in 3D space, it is not surprising that their coalescence would resemble the 2D SHS problem more than the 3D problem. Hill has recently suggested that, for RFM purposes, SHS should be expressed in terms of an optimal noninteger dimensionality, which reflects underlying structures of this type [54].

It is not our purpose to delve too deeply into the complex subject of microstructural effects upon detonation; however, this example serves to give a feeling of how and why they are important, and how detailed pictures such as those of Figure 6b and Figure 9 can fundamentally affect one’s views on modeling. One simply cannot look at these pictures and propose spherical void distributions.

### 4.3. Future Work with Non-Phase-Segmentable HE

It was noted earlier that optical microscopy is often used to characterize HE microstructure and that micrographs are occasionally digitized or otherwise used as reference points for synthetic microstructures in simulations. Another useful finding of this present work is that almost all of the HE samples were at least able to be visualized at a similar quality to most optical microscopy. Grain boundaries could be observed by eye, so grain size and distribution of sizes could be manually calculated from µCT slices using stereological approaches in a similar way that micrographs would be used. A digitization or manual tracing of at least the larger grains should be successful. This demonstrates that the current µCT capability is at least as useful as conventional optical microscopy while requiring no sample preparation, which is important both in terms of time and effort and also for eliminating the possibility of damaging the microstructure when polishing. It is also important to note that polishing a 2D surface removes any voids measurable at the surface, so estimates of void content from microscopy may be inaccurate. As mentioned earlier, even manual or semiautomatic microstructural characterization is useful for determining relationships between the structure of a given HE to all manner of reaction and detonation phenomena.

As technology in both image collection and image analysis improves, as seen in the dual-energy imaging technique demonstrated here, we will likely be able to segment the various constituents of each of the PBX materials, though the melt-cast materials will remain difficult due to the density similarities of the constituents. Future approaches could include doping one material with a higher Z element to enhance the contrast, though obviously this has drawbacks in terms of changing the other sample properties (i.e., doped materials may not consolidate identically during pressing and will affect mechanical properties). Future PBX materials with naturally different elemental composition would also lend themselves to a more accurate 3D measurement of the microstructural differences between crystals and the binder.

## 5. Conclusions

Characterization of the microstructure of several classes of high explosive materials was performed using state-of-the-art X-ray microcomputed tomographic techniques. Segmentation of these composite materials into their constituent phases was attempted using several techniques and showed great promise for at least a few of the materials. PBX 9502 was able to be segmented so effectively that the measured composition matched reality, while PBX 9501 segmentation was moderately effective. For the other materials, software segmentation could be used to segment voids from solid materials, but segmentation between solid phases was not possible. However, accurate representations of voids and their size distribution should still be useful for certain modeling scenarios even if the solid phases are indistinguishable from each other. Each type of data (full tomographic detail, general microstructural characterization, and void size distribution statistics) should be implementable for a variety of simulations. Microstructural images were of similar quality to typical optical microscopy, providing useful detail while minimizing sample preparation and eliminating the possibility of introducing defects during preparation (e.g., cutting and polishing). Overall, this investigation demonstrated the ability to characterize microstructural features such as voids, cracks, crystal sizes, and crystal agglomeration across a wide variety of relevant explosive materials. Such information is critical for future modeling and simulation efforts.

## Figures and Tables

**Table 1:** High explosive materials studied with µCT.

Sample	Mass Composition (wt%)	Processing Method
HMX-HTPB	80% HMX ^1^ 20% HTPB ^2^	Cast cured as a block, then cut to shape
PBX 9501	95% HMX 5% Plasticized Estane 5703	Slurry formulated to form molding prills, then pressed
PBX 9502	95% TATB ^3^ 5% Kel-F	Slurry formulated to form molding prills, then pressed
PBX 9404	94% HMX 6% Plasticized Nitrocellulose	Slurry formulated to form molding prills, then pressed
LX-14	95.5% HMX 4.5% Estane 5703	Slurry formulated to form molding prills, then pressed
PBXN-5	95% HMX 5% Viton A	Slurry formulated to form molding prills, then pressed
PBXW-14	50% HMX 45% TATB5% Viton A	Slurry formulated to form molding prills, then pressed
Composition B	60% RDX ^4^ 40% TNT ^5^	Melt cast, then pressed
Octol	75% HMX 25% TNT	Melt cast, then pressed
TATB	100% TATB	Pressed

^1^ HMX: cyclotetramethylene-tetranitramine, ^2^ HTPB: hydroxyl-terminated polybutadiene, ^3^ TATB: triamino-trinitrobenzene, ^4^ RDX: cyclotrimethylenetrinitramine, ^5^ TNT: trinitrotoluene.

**Table 2:** Imaging conditions used for the high resolution images of the pressed pellets.

Sample	Images	Time (s)	Pixel Size (µm)	Dual Energy?
HMX-HTPB	4501	30	0.82	
PBX 9501	4501	20	0.76	X
PBX 9502	4501	25	0.76	X
PBX 9404	4501	20	0.76	X
LX-14	4501	20	0.76	X
PBXN-5	4501	25	0.76	X
PBXW-14	3201	13	0.84	
Composition B	4501	25	0.76	X
Octol	2401	15	0.89	
TATB	4501	25	0.76	X

## Supplementary Materials

The following are available online at dx.doi.org/10.25583/1658864—Figure S1: Reconstructed TIFFs of HMX-HTPB; Figure S2: Reconstructed TIFFs of segmented HMX-HTPB; Figure S3: Reconstructed TIFFs of PBX 9501 dual energy SALE image; Figure S4: Reconstructed TIFFs of PBX 9501 after segmenting with Intensity Auto Classification; Figure S5: Reconstructed TIFFs of PBX 9502; Figure S6: Reconstructed TIFFs of PBX 9502 Segmented for binder; Video S1: Slice-through of HMX-HTPB; Video S2: Slice-through of PBX 9501, Video S3: Slice-through of PBX 9502.
